# Evaluation of d-dimer as outcome biomarker in COVID-19 acute respiratory distress patients

**DOI:** 10.1590/S1678-9946202466057

**Published:** 2024-09-23

**Authors:** Simone Magalhães Diniz, Vitor Augusto Queiroz Mauad, Caio Cesar Ferreira Fernandes, Marcelo Rodrigues Bacci

**Affiliations:** 1 Centro Universitario Faculdade de Medicina ABC, Departamento de Medicina Interna, Santo André, São Paulo, Brazil; 2 Centro Universitario Faculdade de Medicina ABC, Departamento de Hematologia, Santo André, São Paulo, Brazil; 3 Centro Universitario Faculdade de Medicina ABC, Departamento de Cardiologia,Santo André, São Paulo, Brazil; 4 Hospital Estadual Mário Covas, Unidade de Terapia Intensiva, Santo André, São Paulo, Brazil

**Keywords:** COVID-19, Acute respiratory distress syndrome, D-dimer, Mechanical ventilation

## Abstract

Acute respiratory distress syndrome is a significant complication in critical care patients. COVID-19 (C19)-associated severe respiratory failure is related to it, and d-dimer rise predicts a worse outcome. To investigate the association between d-dimer and the severity of this respiratory syndrome, we conducted a study in C19 intubated patients. A retrospective, single-center observational study was conducted with 64 C19 adult intubated patients. Strata of d-dimer results between patients was evaluated using survival analysis. Survival was higher in mild respiratory distress patients. D-dimer showed poor sensitivity and specificity in predicting respiratory failure severity. Risk assessment for death showed a higher prevalence of admission d-dimer results (HR 1.335; 95% CI 0.695–2.564). Our sample confidently represented the medical profile of C19 severe patients. Sepsis development in C19 is associated with the inflammatory storm in respiratory distress syndrome. As the receiver operating curves show, the increase in d-dimer results is consistent with inflammation rather than a prognostic biomarker. As expected, severe respiratory distress patients presented higher mortality. In summary, d-dimer results are not associated with the prognosis of C19 respiratory distress syndrome patients.

## INTRODUCTION

Acute Respiratory Distress Syndrome (ARDS) is a significant COVID-19 (C19) pulmonary infection complication. It develops in 42% of patients with pneumonia and 60 to 80% of respiratory infected in the intensive unit environment^1^. Since the C19 pandemic outbreak, the leading causes of mortality have included myocardial damage and respiratory and circulatory failures^2^. Despite advances in respiratory pathophysiological knowledge, we still see a lack of expertise in understanding the role of coagulation biomarkers such as serum d-dimer in early-stage respiratory failure^3^. Although C19 ARDS predisposes patients to thromboembolic phenomena^4^, the increase in d-dimer levels is not thrombosis-specific^5^. As such, d-dimer increase has been associated with a C19 worse prognosis regardless of thrombosis, becoming a resource for intensive care unit hospitalization^6^. D-dimer levels as a possible inflammation biomarker were an off-label determiner of patient decision-making the C19 context. Initiating invasive mechanical ventilation (IMV) was one of the vital decisions made based on knowledge of d-dimer results rather than only clinical criteria^7,8^. On the other hand, delayed IMV had a significant variation in the mortality rate of C19 ARDS patients^9^. To test the role of d-dimer, this study evaluated the correlation and mortality of d-dimer levels in C19 respiratory failure patients.

## MATERIALS AND METHODS

### Study population

A retrospective study was conducted in 2022 in an intensive care unit of a Brazilian hospital during the first C19 pandemic wave. Inclusion criteria consisted of patients with confirmed C19 and ARDS at intensive care unit admission. RT-PCR confirmed the C19 diagnosis. Exclusion criteria encompassed patients already participating in a clinical trial, with current or previous cancer diagnosis, those with Chronic Obstructive Pulmonary Disease (COPD) exacerbation, those who had AIDS and were taking immunosuppressive drugs, those who had acute pulmonary edema or acute asthma, or those who were already on IMV at intensive care unit’s admission.

The variables studied were obtained from the first day of mechanical ventilation and included age, gender, occurrence of acute kidney injury, smoking status, serum d-dimer, ventilatory parameters (arterial oxygen pressure – PaO2), oxygen-inspired fraction (FiO2), positive end-expiratory pressure (PEEP) and saturation oxygen (SatO2). Due to the impossibility of detecting d-dimer levels above 50,000 ng/mL using Enzyme-Linked Fluorescent Assay (ELFA), all the results above this level were reported as 50,000 ng/mL. The manuscript followed the STROBE guidelines for observational studies.

### Diagnosis and classification of ARDS

Patients were diagnosed with ARDS according to the Berlin definition by the European Society for Intensive Care: 1) Acute onset of respiratory symptoms; 2) Presence of bilateral infiltrate on chest imaging, in which pulmonary edema cannot be fully explained by heart disease or fluid overload; and 3) Hypoxemia, classified into three categories of severity: 1) Mild: 200 < PaO2/Fio2 ≤ 300; 2) Moderate: 100 < PaO2/Fio2 ≤ 200; 3) Severe: PaO2 < 100^8^.

### Statistical analysis

Outcome was compared between the three groups using the Kaplan-Meier method. D-dimer results were plotted across ARDS severity status (mild, moderate, and severe) patients as a continuous variable and compared using the Kruskal-Wallis test^9^. D-dimer results were divided according to the first day of hospitalization (initial), the average result during hospitalization, and the higher peak and showed in a scatter plot according to the Steel-Dwass-Critchlow-Fligner procedure^9^, a post hoc analysis made after a non-parametric evaluation to avoid error inference in reduced samples.

Impact of d-dimer on overall survival was evaluated by cox proportional hazards model in a multivariate analysis using the following variables: obesity, history of COPD or asthma, presence of a cardioembolic event, and smoking status.

All analyses were made with the XLSTAT 2021 statistical software considering an alpha error of 5%. The study was approved by the local ethics committee. All patients consented to access to the medical charts.

## RESULTS

**Figure 1:**
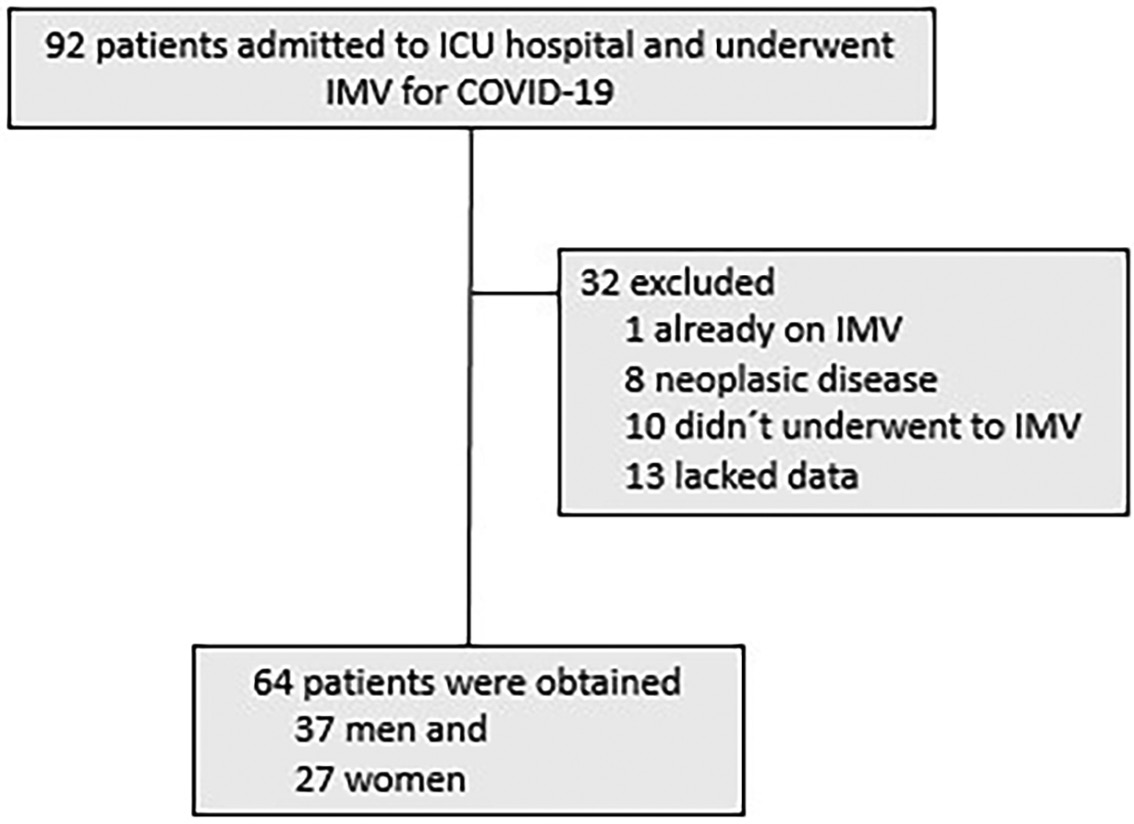
Participant flowechart. ICU = Intensive Care Unit; IMV = Invasive Mechanical Ventilation.

Study sample initially included 96 patients, resulting in 64 participants in the final analysis after evaluation of the exclusion criteria (Figure 1). The sample had 57% men and a higher proportion of patients with hypertension or diabetes. Few patients had acute kidney injury at intensive care unit admission. Mortality across the sample resulted in a ratio of 81.2% of the sample. The sample had a small percentage of patients with obesity (17.19%) defined by a BMI above 30 and diabetes (28.13%). Hypertensive patients represented 51.6% of the sample, and only 9.3% of patients were admitted with kidney damage or known chronic kidney disease (Table 1).

**Table 1:** Clinical and demographic characteristics of patients.

CHARACTERISTIC	FREQUENCY (%)	MEDIAN
N	64	
Age (years)		65
Gender (N)		
Male	37 (57.81%)	
Female	27 (42.19%)	
Kidney damage (N)	6 (9.38%)	
Medical history (N)		
Hypertension	33 (51.6%)	
Diabetes	18 (28.13%)	
MI / Stroke	13 (20.31%)	
Smoking	8 (12.5%)	
Obesity	11 (17.19%)	
COPD / Asthma	3 (4.69%)	
No comorbidities	11 (17.19%)	
D-dimer (ng/mL)		2259,35
PaO2/FiO2 (N)		121,67
Non-ARDS	7 (10,94%)	
Mild ARDS	9 (14,06%)	
Moderate ARDS	21 (32,81%)	
Severe ARDS	27 (42.19%)	
PEEP (cmH2O)		10
SATO2 (%)		96
Outcome (N)		
ICU discharge	12 (18.75%)	
Death	52 (81.25%)	

MI = Myocardial Infarction; BMI = Body Mass Index; COPD = Chronic Obstructive Pulmonary Disease; PaO2 = Partial Pressure of Oxygen in Arterial Blood; FiO2 = Inspired Oxygen Fraction; PEEP = Positive End Expiratory Pressure; SATO2 = Arterial Oxygen Saturation.

Evaluation of cox multivariate regression model found no correlation between ARDS severity and the sample’s previous medical history (Table 2).

**Table 2:** Cox multivariate regression model, with relative risk and 95% confidence interval for change in higher, average, and initial d-dimer.

PARAMETER		HR	95% CI			HR	95% CI			HR	95% CI	
	Higher d-dimer >2800	0.915	0.476	1.757	Average d-dimer > 2150	1.055	0.535	2.081	Initial d-dimer > 2000	1.335	0.695	2.564
Moderate ARDS		2.896	0.333	25.159		2.705	0.326	22.460		2.745	0.329	22.940
Severe ARDS		5.459	0.636	46.855		5.035	0.614	41.291		4.892	0.594	40.308
Age > 65 years		1.397	0.682	2.863		1.395	0.682	2.855		1.413	0.691	2.889
Kidney damage		1.445	0.757	2.758		1.453	0.762	2.771		1.476	0.769	2.832
Hypertension		0.886	0.446	1.759		0.864	0.437	1.709		0.825	0.418	1.629
Diabetes		0.943	0.454	1.958		0.942	0.456	1.947		0.899	0.435	1.856
BMI >30		1.035	0.429	2.498		1.004	0.411	2.451		0.996	0.412	2.409
COPD / Asthma		1.554	0.433	5.571		1.650	0.460	5.924		1.803	0.507	6.409
MI / Stroke		1.811	0.770	4.257		1.833	0.777	4.326		1.877	0.791	4.452

HR = Hazard Ratio; CI = Confidence Interval; BMI = Body Mass Index; COPD = Chronic Obstructive Pulmonary Disease; AMI = Acute Myocardial Infarction; CVA = Cerebrovascular Accident.

**Figure 2:**
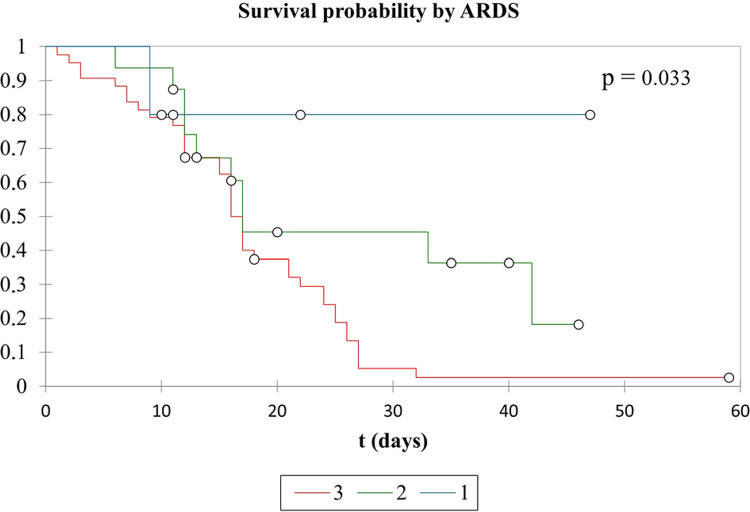
Kaplan-Meier curve in COVID-19 ARSD patients: 1) Mild ARDS; 2) Moderate ARDS; 3) Severe ARDS. ARDS = acute respiratory distress syndrome.

Survival analysis showed a worse prognosis for severe ARDS C19 patients. Kaplan-Meier analysis showed that the median survival in severe ARDS patients occurred on day 16 of IMV and day 17 for the rest of the sample. When considering only patients with mild ARDS, the previous median survival day was not observed considering IMV time. The difference between survival rates had a p-value of 0.033 (Figure 2).

**Figure 3:**
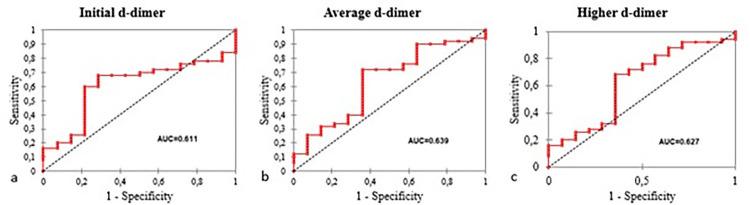
ROC curves of d-dimer: a) initial d-dimer; b) average d-dimer; c) higher d-dimer for the prognostic marker in study subjects.

Evaluation of d-dimer as a prognostic marker was performed using a Receiver Operating Characteristic (ROC) curve to determine the Area Under the Curve (AUC) results (Figure 3). Initial d-dimer strata had an AUC of 0.611 with a 68% sensitivity and 89.5% specificity. Average d-dimer strata was performed with an AUC value of 0.639, a sensitivity of 72%, and a specificity of 64.3%. Higher d-dimer strata showed an AUC value of 0.627, a sensitivity result of 68% and a specificity of 64.3%.

**Figure 4:**
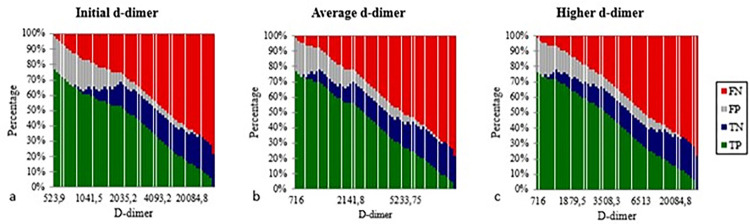
Severity of ARDS and d-dimer results: a) initial d-dimer; b) average d-dimer; c) higher d-dimer. TP = True positive; TN = True negative; FP = False positive; FN = False negative.

Figure 4 shows a better understanding of d-dimer results distribution according to ARDS severity.

## DISCUSSION

This retrospective, observational, intensive care unit, single-center, C19 ARDS–correlated study evaluation of d-dimer results as a prognostic biomarker showed low sensitivity and specificity and no association with patients’ previous medical history in this cohort.

During the C19 pandemic first wave, little knowledge was available to understand the worst risk factors to evaluate patient prognosis. Our study sample fulfilled the main known risk factors for severe C19 with a higher proportion of patients with MI, stroke, hypertension, and diabetes. These characteristics are like the findings of Chang *et al*.^10^, who evaluated C19 patients admitted to the intensive care unit where patients with worse prognosis had hypertension, obesity, and diabetes. Mechanical invasive ventilation increased the severity of C19 patient management. This condition is corroborated by several studies, which offer a higher percentage of males affected by C19 and IMV with higher mortality when IMV occurs^10,11^. Our study sample consisted of patients with a median age of 65, and being critical, we observed a high mortality rate (81.25%). This picture is like those observed by Lim *et al*.^11^, who found a variation in the mortality rate between 47.9% in patients under 40 years and 84.4% in older patients. The researchers also found a direct relation between mortality and aging rather than only correlating with severity strata^11^.

When mortality was assessed according to ARDS strata, we found a significantly higher survival rate for mild ARDS patients than the other ones. Additionally, moderate, and severe ARDS at the beginning of IMV were most related to worse prognosis, followed by thromboembolic events. A similar analysis of ARDS patients’ severity and paO2/FiO2 results stated that the lower this ratio is, the worse the survival^12,13^. Grasselli *et al*.^14^ described that the PaO2/FiO2 ratio increase was associated with improved survival during ICU admission. Getting patients to receive IMV at PaO2/FiO2 >200 could be an alternative for C19 treatment effectiveness. ARDS categories were also evaluated with d-dimer^15,16^. Despite increased d-dimer levels among patients, their values showed no significant difference between the three ARDS strata^15,16^. A slight difference occurred among the AUC strata results: the highest value was observed in the medium d-dimer values (AUC=0.639). Sensitivity was higher (72%) for the strata with the highest d-dimer values, and specificity followed the same pattern, but for the initial d-dimer values collected (89.5%). A recent systematic review evaluated d-dimer results for mortality prediction in critical patients, showing a sensitivity of 75% and a specificity of 83% in a 4,468 patients sample^17^.

Conversely, Toth *et al.* ^18^ conducted a systematic review with meta-analysis to evaluate d-dimer levels among C19 and non-C19 ARDS patients^18^ and found no difference in d-dimer levels among groups but lower mortality in the non-C19 ARDS strata, suggesting an impact of the viral infection. Although the sensitivity and specificity results from this and other studies are differ, they indicate that blood d-dimer is a good sign of inflammation in this group of patients.

Our results might also impact patient follow-up for the long C19 syndrome^19^. Critical care patient survivors have a higher risk of developing long-term C19 due to virus exposure and inflammation during hospitalization^19^. Moreover, d-dimer had a low impact on mortality prediction; nevertheless, it is still a high-quality marker of thromboembolism, demanding supplemental thrombosis investigation^20^.

Study strengths include providing a real-world situation regarding the request for biomarkers for C19 ARDS patients in intensive care. We could only measure d-dimer levels to 50,000 ng/mL due to lab kit limitation; however, there will not be a difference in findings in these levels. Investigators did not interfere in physicians’ treatment decisions per the study’s observational nature. However, its single-center nature makes generalization of the findings difficult. Patients’ nature was mainly clinical due to the cancelation of elective surgeries due to the pandemic. Data was analyzed from a specific part of the year to understand the nature of this biomarker in this scenario. Perhaps a complete whole-year evaluation or for the previous ones could provide more information. The study also provides a clue for the long C19 syndrome in high-risk patients; however, we understand the existence of a low-impact modification in d-dimer sensitivity and specificity results even with a higher sample.

## CONCLUSIONS

In conclusion, evaluating d-dimer sensitivity and specificity in C19 ARDS patients regarding strata presented poor results in prognosis prediction. Patients with severe C19 ARDS presented higher mortality than moderate or mild cases. Future directions involve finding more accurate early inflammation biomarkers to guide the decision tree, leaving d-dimer levels to a non-specific inflammatory evaluation biomarker.
